# Comparison of the Finite Element Method and Rigid Finite Element Method During Dynamic Calculations of Steel–Concrete Composite Beams Based on Experimental Results

**DOI:** 10.3390/ma17246081

**Published:** 2024-12-12

**Authors:** Małgorzata Abramowicz, Agnieszka Pełka-Sawenko

**Affiliations:** Faculty of Civil and Environmental Engineering, West Pomeranian University of Technology in Szczecin, Al. Piastów 17, 70-310 Szczecin, Poland

**Keywords:** dynamic test, composite beam, 3D finite element model (FEM), 3D rigid finite element model (RFEM), modal parameters

## Abstract

Dynamic analysis of structures is a key challenge in structural engineering, especially in choosing effective and accurate numerical methods. Steel–concrete composite structures, commonly used in bridges and floors, require calculations of dynamic parameters to ensure safety and comfort. Few studies compare the effectiveness of the finite element method (FEM) and the rigid finite element method (RFEM) in the dynamic analysis of such structures. This study fills this gap by comparing the methods using experimental results. FEM and RFEM models were developed using Abaqus, Python, and Matlab. The main parameters were identified, i.e., the Young’s modulus of the concrete slab (E_C_) and the stiffness of the connection (K_x_, K_RX_, K_v_, K_h_). Both methods closely matched the experimental results. The RFEM matched natural frequencies with 2–3% deviations, while the FEM showed 3–4% deviations for the torsional, axial, and first three flexural frequencies. The RFEM reduced the computation time by about 65%, making it suitable for large-scale applications. The FEM provided a finer resolution of local effects due to its higher element density. The results can be applied to the design of bridges, floors, and other structures under dynamic loads. It will also provide the authors with a basis for developing structural health monitoring (SHM).

## 1. Introduction

### 1.1. Status Review of the Issue

Composite structures consist of several elements with different material parameters. The basic elements of steel–concrete composite structures are made by joining a steel section with a reinforced concrete slab, which is a very economical solution [1]. Due to the steel and concrete parameters, in the case of free-supported systems, it is important to use the steel elements as the tension part and the reinforced concrete slab as the compression part. In composite structures, the type and the stiffness of the connection is very important [2,3]. Very often, the connection is created using steel studs placed along the beam and embedded in a reinforced concrete slab [4]. Such elements are used as the main beams of road bridges, pedestrian crossings, or as elements of ceilings of public buildings and industrial facilities. Understanding their dynamic behavior for load-bearing capacity or user comfort is an always-present issue [5,6,7,8]. The vertical bending vibrations of objects are most often analyzed, but equally important are torsional vibrations, which are in the same frequency band [9,10]. Loads are very often unsymmetrically distributed, and we obtain bending and torsional vibrations in structures. Due to the role of dynamic loads applied to bridge girders and the requirements of standards for the control of floor vibrations, a dynamic analysis of such structures is necessary.

A reliable computational model is essential both for the engineer’s and the scientist’s work [11,12]. The complexity of a model depends on the scope and precision of analysis. Advanced computer technologies can be used to develop complex computational models and to conduct detailed analysis. The most important thing is that the work of the model should reflect the work of the research object [13]. The composite beam model can be defined in several ways [2,4]. The model can be defined as continuous or discrete. We can develop a model with rigid or deformable elements. There is a possibility to use both, and then, we receive hybrid elements [14,15]. The identification process of one-dimensional continuous models of steel–concrete composite beams is presented in [16]. The identification of two model parameters showed a good match of the axial vibration forms and the first four flexural forms; in higher forms the differences were 20–50%.

A dynamic analysis of composite beams based on continuous models was also conducted [17,18]. In the last paper, three different continuous models of composite beams were defined. The model based on the Timoshenko beam theory turned out to be the most accurate. This model has enabled a very high natural frequency compatibility between the model and experimental research.

In the paper [19], the concept of a 2D discrete model in the convention of the rigid finite element method (RFEM) and the results of experimental dynamic tests were presented. The authors showed that the RFEM can be an effective tool for modeling the steel–concrete beam. The rigid finite element method is a method already established in the 1970s [20]. Over the years, researchers have used it to analyze the structural vibrations in the shipbuilding industry, offshore structures, or construction [21]. The main advantage of the method is the compatibility of the calculated results to experimental findings, with a relatively low number of degrees of freedom.

Analyses of steel–concrete composite beams are very often carried out using the finite element method (FEM). In [22], the authors analyzed the dynamic properties of three steel–polymer concrete composite beams. The beams consisted of steel pipes filled with polymer concrete. The results of the experimental tests with the FEM model analysis showed high running properties. The results of the experimental studies with the FEM model analysis showed high similarity. The spatial FEM model was used to analyze the nonlinear work of beams in bending, and the shear was modeled [23]. The results of the developed models were compared with the results of experimental tests. The proposed FEM model predicted the load limit with sufficient accuracy. In [24], the authors also proposed a spatial, non-linear FEM model of steel and concrete composite beams. The authors analyzed the effect on the bearing capacity of steel–concrete composite beams bonded by adhesive. They determined the properties of the beams depending on the properties and thickness of the adhesive layer. The results of the analysis are compared with the experimental studies.

A reliable numerical model is necessary for every study and can contribute to the development of structural health monitoring (SHM). The concept of SHM includes many techniques for monitoring the condition and preventing damage in engineering structures. Modal analysis is often used to develop various SHM methods [25,26,27,28]. Very interesting analyses were presented in the two-part article [29,30]. In the first part [29], experimental tests were presented for four composite beams. These beams were tested undamaged and damaged. The results of the experimental studies carried out on composite beams with a free beam scheme were presented. The analysis compared the obtained frequencies and modes of free vibrations for the composite beams before and after the failure. In the second part [30], the improved one-dimensional model (compared to the analyses from the article [16]) was presented. The stiffness of the connection was additionally analyzed in both directions: parallel and perpendicular to the beam axis. The results obtained from the numerical models were compared with the results from the experimental studies. A high similarity of the results for the undamaged and damaged models was presented. Another study [31] considered the modeling and estimation of the parameters of composite beams with a partially damaged connection at the end of the beam. The authors developed two models, one was based on the Euler beam theory and the other was based on the Timoshenko theory. Damage was confirmed in the eigenvectors analyzed.

The SHM using non-destructive damage detection methods requires the preparation of a reliable numerical model. Therefore, before the damage detection analysis itself, it is necessary to develop a reliable numerical model (of the undamaged structure), validated based on the results of experimental tests conducted on the real model. The current analysis is part of a larger project to enable damage detection in steel–concrete composite beams [28,32].

The continuous development of computer technology allows modeling and analyzing more and more complex elements and phenomena occurring in them. The choice of the computational method to be used during the analyses depends on the knowledge and experience, as well as the planned further analyses, model expansion or scope of the study. A review of the literature has revealed few scientific papers that directly compare the results of experimental modal analysis at the same time as the results of numerical models made in the convention of the FEM and RFEM methods. Both RFEM and FEM methods have been successfully applied in various research studies. Comparisons of FEM and RFEM methods can be found in several scientific papers [22,33]. RFEM models have been verified in dynamic analyses of offshore lattice jib cranes [34] as well as in the modeling of composite steel–polymer concrete machine tool frames [33]. The results of this study can find application in civil engineering, especially in the design of structures subjected to dynamic loads, such as bridges, buildings, and machinery. The comparison of FEM and RFEM methods allows the selection of a more efficient and economically optimal computational method, while maintaining good accuracy of the results, which is crucial in terms of saving time and resources in the design process. For the purposes of the planned research, the two computational methods were compared in terms of modeling, and obtained results in a dynamic analysis, verifying the capabilities and limitations of both methods in steel–concrete composite beams.

### 1.2. Analysis Fundamentals

The process of determining the dynamic parameters of the numerical model was based on the following formulas and transformations (1)–(5) according to [35]. The general notation of the equation of motion can be presented according to the formula:

(1) $$M\ddot{q}\left(t\right)+C\dot{q}\left(t\right)+Kq\left(t\right)=P(t),$$

where M—the mass matrices, C—the damping matrices, K—the stiffness matrices, $\ddot{q}(t$)—the vector of displacement, $\dot{q}(t$)—the vector of velocity, q(t)—the vector of acceleration, and P(t)—the vector of force.

For the free vibrations problem, damping can be omitted, and Formula (1) can be presented in the form:

(2) $$M\ddot{q}(t)+Kq(t)=0,$$

The solution of the equation of motion (2) is as follows:

(3) $$q\left(t\right)=\phi cos(\omega t+\varphi ),$$

where ϕ—the vector of the form of vibrations, ω—the free vibration circular frequency of the system without damping, and φ—the phase angle.

The equation, after substituting the Equation (3) to (2), is as follows:

(4) $$\left(K-\omega^{2}M\right)\phi =0,$$

A non-trivial solution to Equation (4) exists if

(5) $$det\left(K-\omega^{2}M\right)=0,$$

The solution of Equations (4) and (5) is N eigenvalues $\omega_{1}^{2}$, $\omega_{2}^{2}$, …, $\omega_{N}^{2}$, which correspond to N eigenvectors $\phi_{1}$, $\phi_{2}$, …,$\phi_{n}$, $\phi_{N}$. By writing the vectors $\phi_{n}$ in the following columns of the matrix, we will create a matrix of eigenvectors ϕ.

During the creation of the numerical models, most parameters of steel–concrete composite beams were established through the inventory and material data, while others were established through the identification process. In both cases, the vector of four decision values was as similar as possible and can be given by the following:

(6) $$x_{FEM}=[K_{x},\;K_{v},\;K_{h},\;E_{C}],$$

(7) $$x_{RFEM}=[K_{R,X},\;K_{v},\;K_{h},\;E_{C}],$$

where $K_{x}$—the substitute stiffness of connection in the normal direction (along the *x*-axis), $K_{v}$—the substitute stiffness of connection in the tangential direction (along the *y*-axis), $K_{h}$—the substitute stiffness of connection and in the normal direction (along the *z*-axis) to the connection plane, $K_{R,X}$—the substitute stiffness of connection in the rotational stiffness, and *E_C_*—the substitute dynamic longitudinal modulus of elasticity of the reinforced concrete slab.

The optimization process, relative to earlier work [36,37], was expanded to include an analysis of additional forms of vibration, and was carried out in two stages. The criterion of the first stage $S_{I}$ was the best fit of the first frequency of distortional natural vibration (*d*). This criterion made it possible to determine the substitute stiffness of the connection in the normal direction $K_{x}\;$(along the x axis), hence

(8) $$S_{I}={\left(\frac{f_{exp}^{d}-f_{num}^{d}}{f_{exp}^{d}}\right)}^{2},$$

where $f^{d}$—the first distortional vibration mode, *exp*—results obtained from experimental data, and *num*—results obtained from discrete model.

In the second stage, the criterion $S_{II}$ was the best fit of the first five frequencies of flexural natural vibrations (*vf*), the first two frequencies of torsional natural vibrations (*t*), and the fundamental first axial natural vibration (*a*) forms. These calculations allowed for the determination of other model parameters. The criterion $S_{II}$ can be written as follows:

(9) $$S_{II}=\sum_{i=1}^{m}{\left(\frac{f_{exp}^{i\_vf}-f_{num}^{i\_vf}}{f_{exp}^{i\_vf}}\right)}^{2}+\sum_{i=1}^{n}{\left(\frac{f_{exp}^{i\_t}-f_{num}^{i\_t}}{f_{exp}^{i\_t}}\right)}^{2}+{\left(\frac{f_{exp}^{i\_a}-f_{num}^{i\_a}}{f_{exp}^{i\_a}}\right)}^{2},$$

where *S*—the sum of squares of relative deviations of numerical (*num*) and experimental (*exp*) frequencies *(f*) of the first five modes of flexural vibrations (*vf*), the two torsional vibrations (*t*) and the fundamental mode of axial vibration (*a*). Both the first distortional vibration mode and the torsional vibrations have not yet been implemented in the currently developed FEM models of composite beams [36,37]. A review of the literature indicates that they are equally important in the analyses [12,32]. The inclusion of these additional forms of vibration in the optimization of steel–concrete composite beams enriches the numerical models and enables additional analyses of spatial systems.

When the optimization process was completed, the vectors vibration forms for the model were verified, calculated with the Modal Assurance Criterion (MAC) [38,39]. In the papers [40,41,42,43], the authors used the MAC to verify the credibility of the results. The MAC index is scalar and expresses the relationship between the compared vibration modes received during the numerical calculation and the results received in experimental tests. By analyzing the vectors of various forms of vibration, a matrix is created. If the value of the MAC coefficient is between 0.8–1.0 and additionally lies on the main diagonal of the matrix, it means that the analyzed vectors are similar. The MAC indicator can be shown as follows:

(10) $$MAC=\frac{{\left|\phi_{exp}^{T}\phi_{num}\right|}^{2}}{\phi_{exp}^{T}\phi_{exp}\cdot \phi_{num}^{T}\phi_{num}}$$

where $\phi_{exp}$—the modal vector for the results of the experimental research and $\phi_{num}$—the modal vector for the results of the analysis of the calculation model.

**Figure 1:**
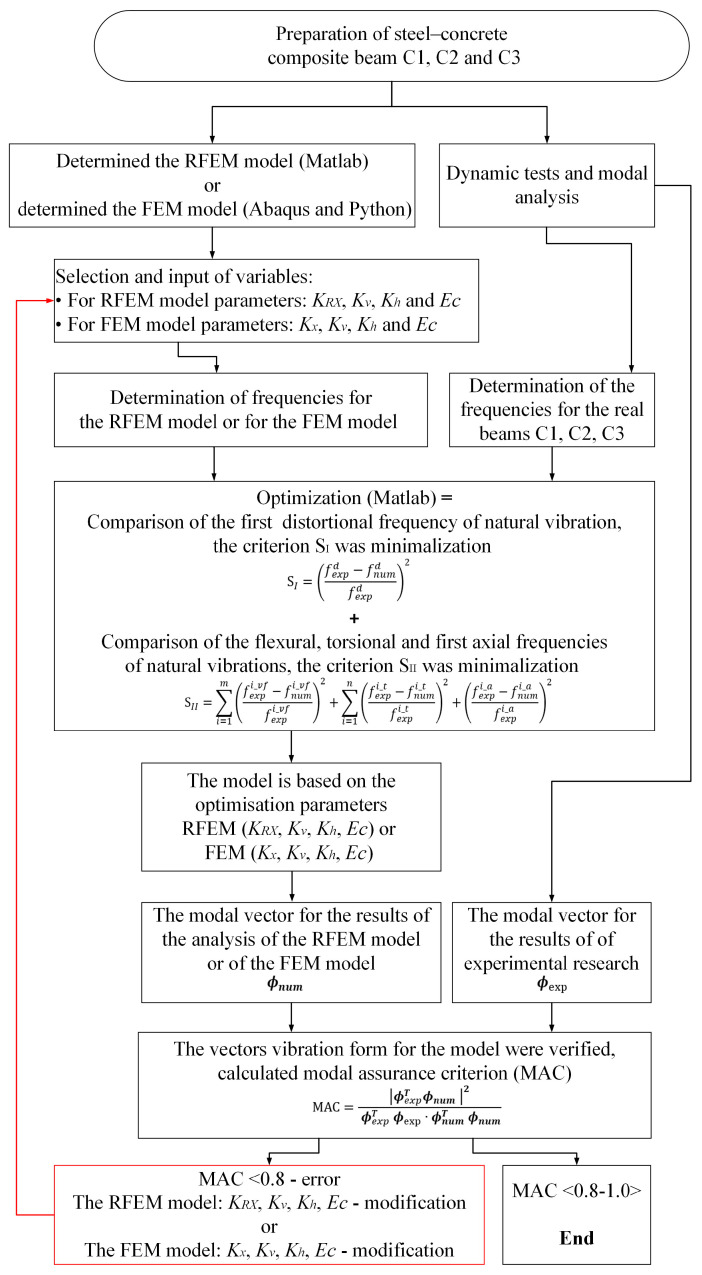
The block diagram for the methodology used to determine the parameters for the RFEM model or for the FEM model.

The methodology used to determine the parameters for the RFEM and the FEM models is presented in Figure 1. The block diagram describes the process for both the RFEM and FEM simultaneously.

## 2. Materials and Methods

### 2.1. Steel–Concrete Composite Beams

**Figure 2:**
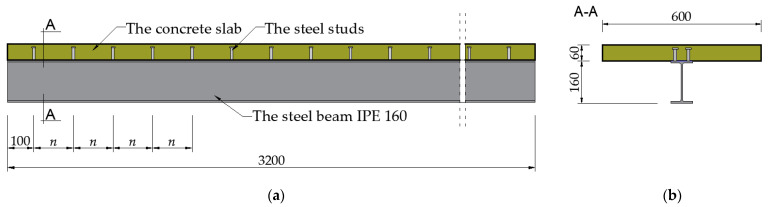
The analyzed steel–concrete composite beam: (**a**) longitudinal cross-section and (**b**) cross-section.

The 3.2 m long composite beam consisted of the reinforced concrete slab (600 × 60 mm) and the steel structural profile IPE 160. The structural steel profile was made from S235JRG2 steel according to [44], with a Young’s modulus of 210 GPa, a transverse deformation factor of 0.3, and a yield strength of 235 MPa. The slab was made of C25/30 concrete in accordance with [45], with the standard specified basic parameters, i.e., a Young’s modulus of 30 GPa, a transverse deformation factor of 0.2, and compressive strength equal to 25 MPa for the cylindrical specimen and 30 MPa for the cubic specimen. Grade A-III (34GS) 8 mm diameter reinforcing bars were used in the slab. The composite beam used KÖCO type SD stud connectors with a diameter of 10 mm and a height of 50 mm. The pair spacing (*n*) of the connectors along the length of the beam was 100 mm, 150 mm, and 200 mm for beams C1, C2, and C3. The scheme of the beam is shown in Figure 2.

It should be mentioned that each beam, before the impulse test, was subjected to an inventory with mass determination. Before the identification of the numerical models, some of the parameters of the beams were selected from data in the literature. The density of the concrete was adjusted so that the final mass of the numerical model was identical to that of the actual beam. The value of Young’s modulus was determined on the basis of identification and assumes that the stiffness of the slab reinforcement used is included. Table 1 shows the technical data of the materials used in the composite beams.

### 2.2. Dynamic Tests

**Figure 3:**
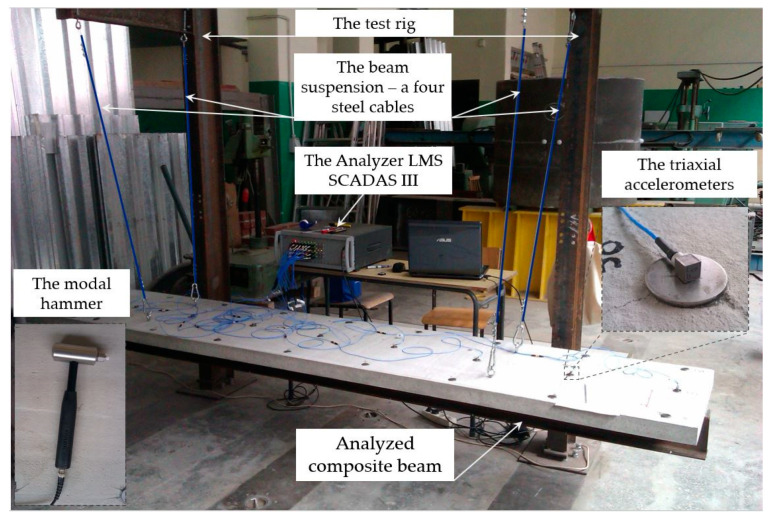
Experimental research site: view of the test rig and analyzed beam in the rig with measurement equipment.

The objective of the experimental study was to ascertain the fundamental dynamic characteristics of the beams under examination, specifically the frequencies and the associated natural vibration modes. This study encompassed the analysis of three composite beams. The beams were tested on a purpose-developed test rig. The test was carried out for a free beam scheme. The test apparatus consisted of two steel columns with brackets; the columns were braced together to prevent rotation. The beams were suspended from the frame using four steel cables with a diameter of 3 mm. During the test, the location of the cables was chosen to coincide with the first-order ties, allowing the beams to be analyzed as free. A schematic of the test set-up is shown in Figure 3.

**Figure 4:**
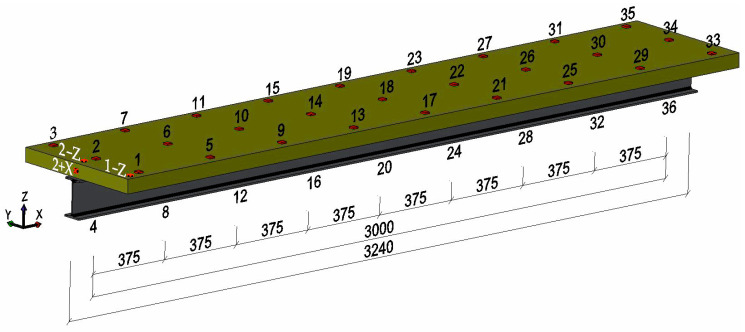
The excitation points 2 + X, 2 − Z, and 1 − Z, and measuring points from 1 to 36.

In the course of the experiment, the following instruments were employed: Nine PCB 356A01 triaxial accelerometers (PCB Piezotronics, Depew, NY, USA), as illustrated in Figure 3. The sensors were affixed to the concrete slab with the aid of a specialized wax provided by the sensor manufacturer. The impulses were investigated with excitation generated at three points: two of these were located on the upper surface of the panel, and the third was situated on the front surface. The excitation points are illustrated in Figure 4. The excitation points are as follows: 2 − Z vertical impact in the middle of the end of the beam, 1 − Z vertical impact on the edge of the beam, and 2 + X horizontal impact on the face of the beam. The various excitation points were used to obtain different vibration modes of the beams.

The vertical flexural mode was obtained using Point 2 − Z. The objective of applying Point 1 − Z was to investigate the vertical flexural and torsional modes. The fundamental axial mode of vibration was obtained using Point 2 + X. The impulse excitation was generated using a 320 g Modal Hammer, ICP, 086D05 impact hammer (PCB Piezotronics) with a medium–hard white plastic insert (084B04) tip (Figure 3). During the course of the tests, vibration accelerations were recorded at a total of 36 fixed points. A total of 27 points were distributed uniformly on the reinforced concrete slab, while 9 points were located on the bottom flange of the steel section, as illustrated in Figure 4. Due to the considerable number of points involved, the study was conducted in stages. In one stage, nine sensors were used to measure the acceleration at different points along the same line. The resulting acceleration responses were recorded using the LMS SCADAS III (Siemenes, Planto, TX, USA). This system was connected to a workstation equipped with a computer-aided system, and the LMS Test Lab package was used to record the signals. The Impact Testing module of the LMS Test Lab package was used for the impulse tests.

The PolyMAX module of the LMS Test Lab program was used for the analysis of the dynamic test results. A stabilization diagram was used to determine the frequencies of interest and subsequently read out the vibration modes for the selected frequencies. The analyses concentrated on the initial five forms of torsional and flexural vibration. Furthermore, the fundamental form of axial vibration and distorted vibrations connected to the flange were also examined. In order to obtain this set of frequencies and shapes, it was necessary to apply different forcing points. The research discussed is described in detail in the article [32]. The experimental results for individual beams are presented in Table 2.

### 2.3. The FEM Model of Steel–Concrete Composite Beams

**Figure 5:**
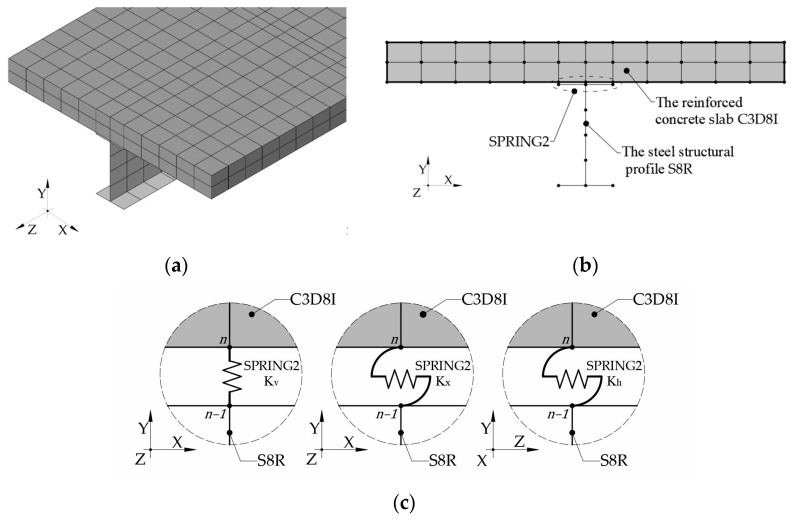
The FEM model of the steel–concrete composite beam: (**a**) the global view, (**b**) the cross-section of the beam, and (**c**) SPRING2 elements.

The model of the steel–concrete composite beam was defined in an Abaqus/CAE 2022 and Python 3.12 environment. The analysis was conducted on the assumption of the elastic behavior of the beams. The spatial arrangement was modeled with, independently, the reinforced concrete slab, the steel beam, and the connection. The approach to modeling steel–concrete composite beams is different. In [46], the authors proposed a system consisting of shell elements, both for the plate and the steel beam. A model with solid elements was proposed in [47,48]. A composite beam model combining solid elements with shell elements was used in [23,49,50]. Solid and beam elements were proposed in [2,4,37,41]. The scheme of the FEM model of the steel–concrete composite beam is presented in Figure 5.

The reinforced concrete slab was modeled using solid elements (C3D8I), while shell elements (S8R) were employed to represent the structural steel IPE 160 profile. Three shells were defined in the model. The first shell defines the web of the beam while the other two define the top and bottom flanges. The thicknesses of these shells were selected in such a way as to ensure that the resulting stiffness and cross-sectional area closely match the actual geometry of the steel section. The distance between the reinforced concrete slab and the steel profile was precisely half the thickness of the shell that defines the top flange of the steel structure. The basic finite element mesh for the reinforced concrete slab was assumed to use twelve elements per width and two elements per height. The basic finite element mesh for the steel beam was assumed to use two elements on the width of the top and bottom flanges and four elements on the web. In the longitudinal direction of the beam, the mesh size was set to 50 mm.

The approach to defining the connection is different and depends on the analyses performed. In works such as [23,36,46,51], the connection was modeled as beam elements connecting the nodes of the elements modeling the concrete slab with the nodes of the steel beam. In works [24,47,48,49,52], a very accurate model of the connecting pins was used, defining them as solid elements. Spring elements modeling the connection were used in works such as [24,36,52]. During the definition of the connection between the reinforced concrete slab and the steel beam, spring elements (SPRING2) were used. Three groups of spring elements were created. The first group of spring elements was responsible for stiffness *K_x_* (the tangential directions to the connections plane, along the *x*-axis), the second group of spring elements was responsible for stiffness *K_h_* (the tangential directions to the connections plane, along the *z*-axis), while the third group was responsible for the interaction between elements in the normal directions to the connections plane—stiffness *K_v_* (along the *y*-axis). The spring elements were used to connect the nodes of the reinforced concrete slab with the nodes of the steel beam. The spacing of the elastic elements along the length of the beam was identical to that of the steel studs in composite beams, with three elements per width being used in the section. The stiffness of SPRING2 elements was determined during the identification.

At this stage of the analysis, the calculation model was defined on the basis of the assumption that the beam has a static diagram with free ends, that its cross-section is constant along its entire length, and that only five bending modes and one axis were analyzed [36,37].

During the identification process, Matlab (R2020b) solvers were used. It was necessary to ensure fully automatic communication between the program in which the steel–concrete beam model was created and the program in which the optimization process was carried out. It was important to assume the initial conditions (starting vector). The assumption of extremely different values from the actual ones disturbed the computational analyses in the Abaqus/CAE 2022 program, because the Matlab (R2020b) program was also accepting negative values. The assumption of negative values resulted in the cancelation of analyses at the level of model verification. The appropriate identification step and selection of the starting point were critical to the successful completion of the identification process. The identification process was conducted two stages, based on the results of the experimental studies. The vector of the decision variables was described by Formula (6). In the first step, the first distortional vibration mode ($f_{1,dist}$) was identified through the minimization of S_I_, as determined according to Formula (8). In the second stage of the analysis, an attempt was made to match the first five frequencies of flexural natural vibrations ($f_{i,flex}$), the first two frequencies of torsional natural vibrations ($f_{i,tors}$), and the fundamental first axial natural vibration ($f_{1,long}$) forms, with the objective of minimizing S_II_, defined by Formula (9).

**Figure 6:**
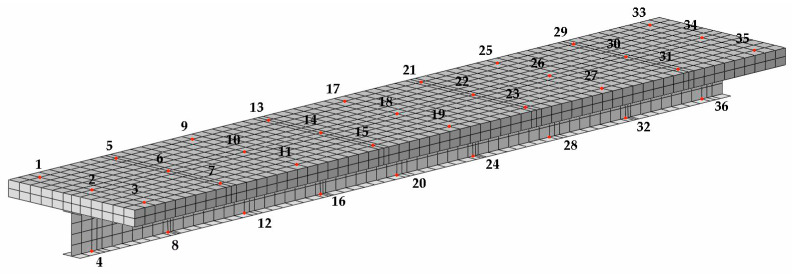
Measurement points for MAC calculations in FEM models.

The verification of each model was conducted through the determination of the Modal Assurance Criterion according to Formula (10). The MAC values were calculated by extracting the mode shapes at specific points. These points were located at the same positions as those used in the experimental tests, as illustrated in Figure 6.

### 2.4. The RFEM Model of Steel–Concrete Composite Beams

A discrete computational model of a composite beam was developed as an alternative to the finite element method using the rigid finite element method. The rigid element method was developed by Kruszewski et al.—a team from Poland [53,54,55]. RFEs are characterized by the mass $m^{\left(i\right)}$ and the mass moments of inertia $J_{X}^{\left(i\right)},J_{Y}^{\left(i\right)},J_{Z}^{\left(i\right)}$, described by a diagonal mass matrix (11) as follows:

(11) $$M^{\left(i\right)}=diag\left[m^{\left(i\right)},\;m^{\left(i\right)},m^{\left(i\right)},J_{X}^{\left(i\right)},J_{Y}^{\left(i\right)},J_{Z}^{\left(i\right)}\;\right],$$

**Figure 7:**
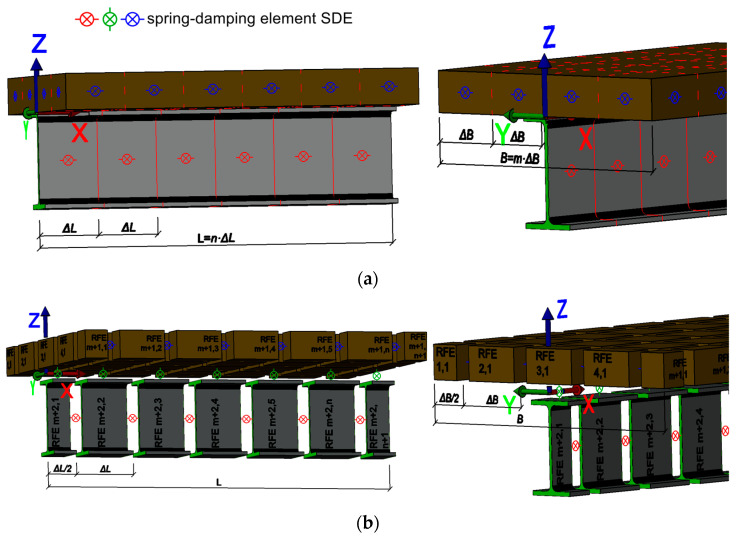
3D rigid finite element model of composite beam: (**a**) primary division into segments and (**b**) secondary division into RFEs connected by SDEs.

Each rigid finite element (RFE) numbered *i* has its own independent reference system $X_{RFE}^{\left(i\right)}$, $Y_{RFE}^{\left(i\right)}$, $Z_{RFE}^{\left(i\right)}$. This system is chosen to coincide with the central inertia system of the RFE in question. The method involves dividing the slab and the beam separately into non-deformable solids called rigid finite elements. Elements like the slab and the beam were divided by length into as many elements. In width, the division was made only for the slab. This division is shown in Figure 7a, which is the primary division. The rigid finite elements are connected between each other by spring-damping elements (SDE). SDEs are characterized by stiffness and damping coefficients. Each SDE with a number *k* has its own independent reference system $X_{SDE}^{\left(k\right)}$, $Y_{SDE}^{\left(k\right)}$, $Z_{SDE}^{\left(k\right)}$. The damping properties can be neglected when solving for the natural vibration of the structure. The properties of the SDE are summarized in two matrices: the first is a stiffness matrix consisting of three translational stiffness coefficients $k_{T,X}^{\left(k\right)}$, $k_{T,Y}^{\left(k\right)}$, and $k_{T,Z}^{\left(k\right)}$ and three rotational stiffness coefficients $k_{R,X}^{\left(k\right)}$, $k_{R,Y}^{\left(k\right)}$, and $k_{R,Z}^{\left(k\right)}$, described by a diagonal stiffness matrix (12); the second is a damping matrix consisting of three translational damping coefficients $c_{T,X}^{\left(k\right)}$, $c_{T,Y}^{\left(k\right)}$, and $c_{T,Z}^{\left(k\right)}$ and three rotational damping coefficients $c_{R,X}^{\left(k\right)}$, $c_{R,Y}^{\left(k\right)}$, and $c_{R,Z}^{\left(k\right)}$, described by a diagonal damping matrix (13). The secondary division is presented in Figure 7b.

(12) $$K^{\left(k\right)}=diag\left[k_{T,X}^{\left(k\right)},\;k_{T,Y}^{\left(k\right)},k_{T,Z}^{\left(k\right)},k_{R,X}^{\left(k\right)},\;k_{R,Y}^{\left(k\right)},k_{R,Z}^{\left(k\right)}\;\right],$$

(13) $$C^{\left(k\right)}=diag\left[c_{T,X}^{\left(k\right)},c_{T,Y}^{\left(k\right)},c_{T,Z}^{\left(k\right)},c_{R,X}^{\left(k\right)},c_{R,Y}^{\left(k\right)},c_{R,Z}^{\left(k\right)}\right],$$

The next step is the displacement transformation process, which aims to relate the strain potential energy of the SDE to the coordinates of the generalized rigid finite elements connected to that SDE. For this purpose, the relationship between the generalized coordinates of the RFE and the attachment coordinates of the SDE to the given rigid finite element has to be determined. In the developed model, the axes of the SDE and RFE are parallel to each other.

The global stiffness matrix **K** is formed from the stiffness matrix $K^{\left(k\right)}$ for single SDEs, the global damping matrix **C** is formed from the damping matrix $C^{\left(k\right)}$ for single SDEs, and the inertia matrix **M** is formed from the mass matrix $M^{\left(i\right)}$ for single RFEs.

**Figure 8:**
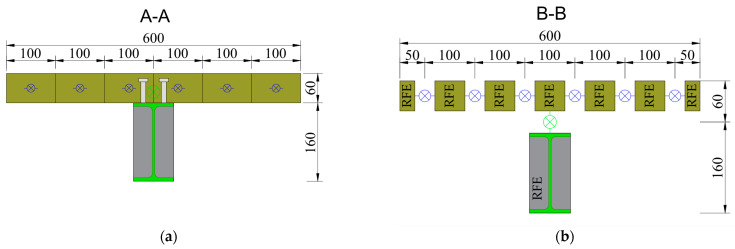
3D modes of RFEM of composite beams—cross-sectional view: (**a**) primary divisions and (**b**) secondary divisions.

An SDE was assigned to each section resulting from the primary division: for the steel section 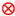, each SDE concentrated the elastic-damping properties of the steel I-beam; for the reinforced concrete section 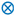, it concentrated the elastic-damping properties of the reinforced concrete slab in the X and Y directions, respectively. Between the resulting SDEs for the reinforced concrete slab and I-beam, separately, rigid finite elements RFE with lengths resulting from the secondary division were inserted. For the edge elements, the element lengths were ΔL/2 in the *X*-axis direction and ΔB/2 in the *Y*-axis direction (for the reinforced concrete slab), while for the others, they were ΔL and ΔB, respectively. The final element completing the model was the SDEs modeling the joint 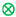 (‘contact point’) between the reinforced concrete slab and the steel I-section. These connect the SES edges of the reinforced concrete slab and the steel I-beam. The final element completing the model were the SDEs modeling the composite (interface) between the reinforced concrete slab and the steel I-section. These connect the RFE edges of the reinforced concrete slab and the steel I-beam. When modeling the composite beam, the primary division in the transverse direction of the slab was made into an even number of sections. This division always results in an odd number of rigid finite elements across the slab in the secondary division, which resulted in the central rigid element being located directly above the rigid element modeling the steel section. For the beams analyzed, the number of elements in the transverse direction for the reinforced concrete slab at primary division was always 6, and at secondary division always 7 (Figure 8).

The stiffness of the composite member was determined by the axial stiffness in the *Z*-axis direction, the coefficient K_v_, the shear stiffness in the *X*- and *Y*-axis directions, and the coefficient K_h_. The designation ‘axial stiffness’ is understood as the loading direction of the steel studs—this is the vertical direction (studs are loaded along their axis). Using the designation ‘shear stiffness’, this is understood as the stiffness of the composite element preventing slippage at the steel–concrete interface. In addition, a parameter of rotational stiffness with respect to the *X* axis is defined, which is denoted K_R,X_. The stiffness matrix **K**_CON_ takes the form of a diagonal matrix of dimension 6 × 6 (14), as follows:

(14) $$K_{CON}=diag\left[K_{h},\;K_{h},K_{v},K_{R,X},0,\;0\right]$$

**Figure 9:**
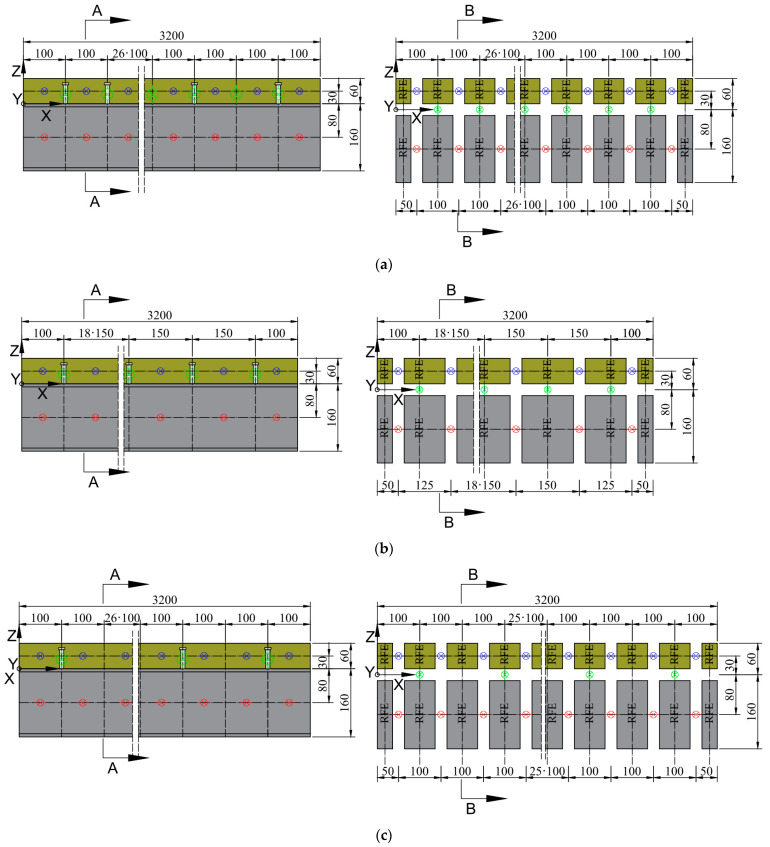
3D modes of RFEM of composite beams—axial view for primary and secondary divisions: (**a**) C1 beam; (**b**) C2 beam; and (**c**) C3 beam.

The primary and secondary divisions for beams C1, C2, and C3 are shown in Figure 8 and Figure 9.

Numerical experiments were carried out to determine the finite element density of the slab in order to determine the necessary number of elements. When determining the partitioning density, the accuracy of the solution, the calculation time, and the position of the composite elements—the steel studs—were taken into account. The number of RFEs and SDEs obtained for the beams analyzed are summarized in Table 3.

## 3. Results

### 3.1. Results from RFEM Calculations

For the RFEM model analyzed, the results are presented in Table 4. The vector of decision variables is presented in Table 5.

**Figure 10:**
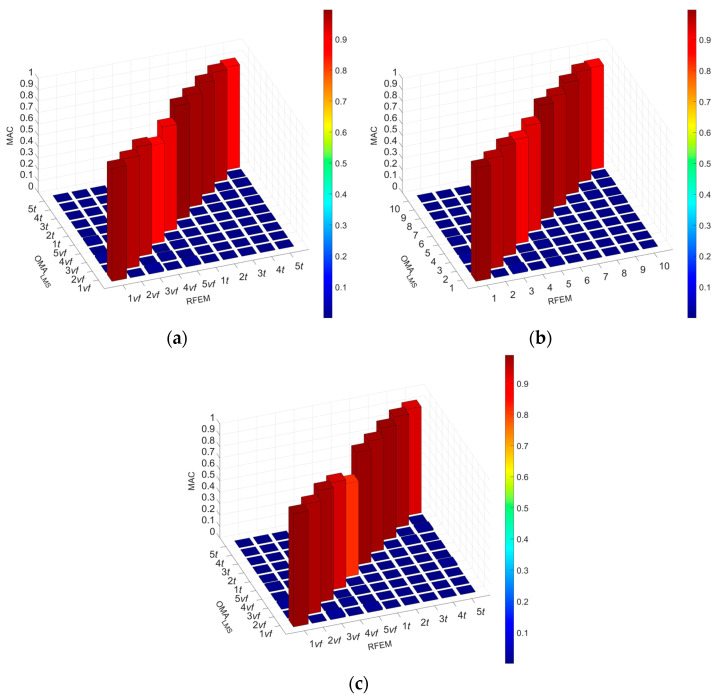
MAC results for RFEM composite beams: (**a**) C1 beam, (**b**) C2 beam, and (**c**) C3 beam.

**Figure 11:**
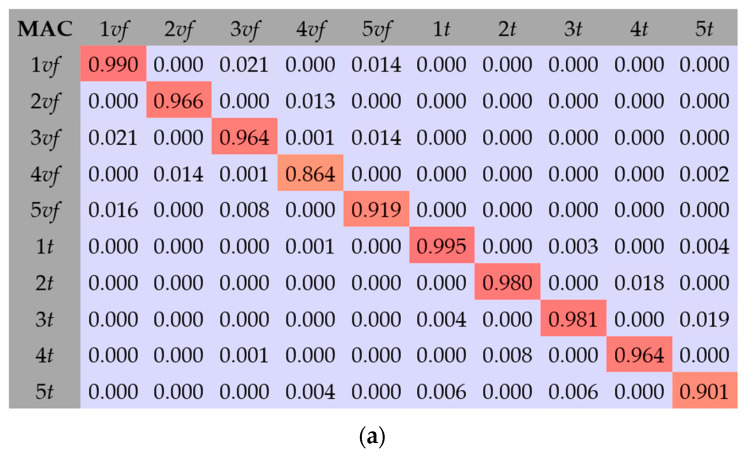

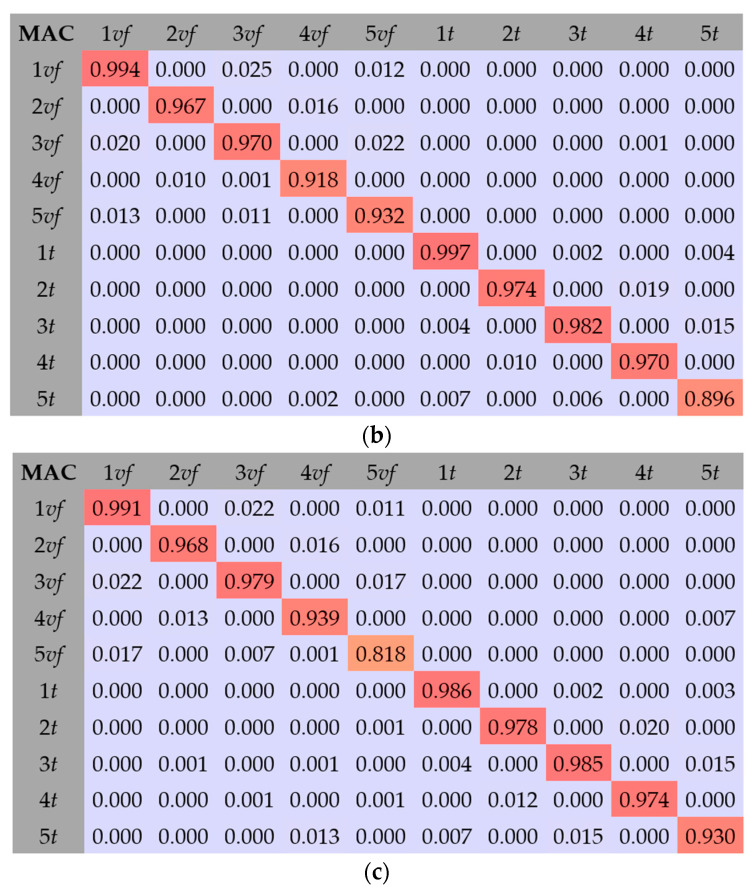
MAC matrix calculations for five flexular and torsional mode shapes for RFEM: (**a**) beam C1, (**b**) beam C2, and (**c**) beam C3.

The comparison of the results of the vibration mode shapes of the model with the results from the experimental tests as MAC is presented in Figure 10 and Figure 11.

### 3.2. Results from FEM Calculations

During the comparison of the FEM and RFEM methods based on the calculation of the steel–concrete composite beams, several types of calculations were performed. The first type of calculation (TC1) presents the results with the vector of decision variables obtained during the RFEM identification calculations. The second (TC2) presents selected results of the identification of the FEM models with some identification conditions. The third (TC3) presents selected results of the identification of the FEM models without any identification conditions. Finally, the fourth type of calculation (TC4) presents the results of an assumed constant, the Young’s modulus of concrete (E_c_), taken from the RFEM identification.

During the first calculations (TC1), an attempt was made to estimate the vector of the decision variables obtained during the RFEM model identification, in order to assess the similarity of the stiffness connection and the natural frequency results. Both methods are characterized by different modeling approaches, making it impossible to generate identical models for a direct comparison. In the numerical models RFEM and FEM, the number of spring elements in the cross-section was different. To estimate the vector of the decision variables, the stiffness of the connection was calculated linearly. In both models, the method of defining the stiffness in the x-direction was also different. For the FEM model, it was necessary to define an elastic element in N/m units; for the RFEM model, one elastic element in the cross-section required using an elastic element with the unit Nm/m. The obtained natural frequencies of the first five vertical flexural (1–5*vf*), the first five torsional (1–5*t*), the first distortional (1*d*), and the first axial (1*a*) vibrations were compared with the experimental results and are presented in Table 6. The vectors of the decision variables are presented in Table 7.

As can be seen, the matching of the obtained results with those from experimental tests for torsional forms is quite correct. Significant differences can be observed when analyzing other vibration modes. It can be concluded that the attempt to linearly calculate the stiffness of the connection between the models proved to be an oversimplification.

During the TC2 calculations, the decision variable vector was estimated by identifying the FEM models. The initial analyses involved identification attempts similar to those in the RFEM model, where the first distortional mode (1*d*) was used to estimate Kx. However, in the FEM model, it was found that just a specific value for matching this frequency could be achieved. Later attempts to increase the stiffness did not improve the matching of this frequency. Therefore, it was decided to carry out identification with constraints. The identification conditions were set so that the program determined the decision variable vector by fitting the first axial vibration mode obtained from the experimental tests. These results are presented in Table A1. In Table A2, the values of the decision variable vectors and the individual identification criteria are presented.

As can be seen, the matching of the flexural vibration modes, the first distortional (1*d*), and the first axial (1*a*) has been significantly improved. For the relatively low identification criterion S, the torsional vibration modes are less well matched compared to those in the TC1 identification. The program estimated a much lower E_C_ than in the case of calculations in the TC1 and, as can be seen, increased the stiffness of the elastic elements. Nevertheless, the required perfect fit was achieved. However, the low E_C_ values prompted the decision to conduct another analysis.

During the TC3 calculations, a separate vector of decision variables was estimated during the identification of the FEM models, without any additional constraints being applied to the program. The results are presented in Table A3. The obtained frequencies were compared with the results of experimental tests. In Table A4, the values of the decision variable vectors and the individual identification criteria are presented.

As can be seen, the results show a high convergence with the experimental results in some places. In the case of optimization without constraints, the results of fitting the natural frequencies for torsional and lower flexural forms are better fitted, while the highest flexible forms of bending forms are worse fitted. The problem was still fitting the first distortion (1*d*) which estimates K_x_. The observed discrepancies in the values of Young’s moduli between the different identifications led the authors to conduct further numerical analyses.

During the TC4 calculations, a separate vector of decision variables was estimated during the identification of FEM models with E_C_ value taken from the RFEM identification, without any additional constraints applied to the program. The results are presented in Table 8. The frequencies obtained were compared with the results of experimental tests. In Table 9, the values of the decision variable vectors and the individual identification criteria are presented.

The assumed value of the E_C_ modulus significantly improved the identification process and significantly reduced the computational time. It can be observed that, at present, only the highest bending vibration mode for beam B2 shows a deviation greater than 7%. The remaining natural frequency shows a high degree of convergence between the models. This stage of the calculations was considered final, and the MAC values for the vibration modes were extracted and compared at this point.

**Figure 12:**
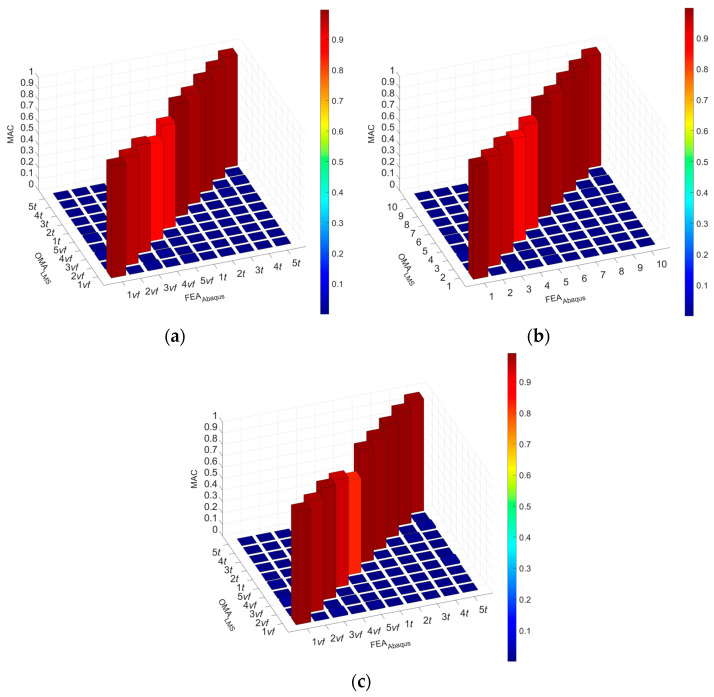
MAC results for FEM composite beams: (**a**) C1 beam, (**b**) C2 beam, and (**c**) C3 beam.

**Figure 13:**
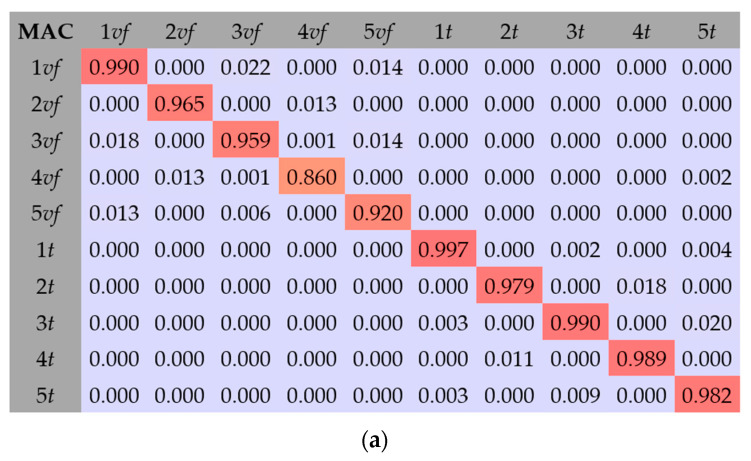

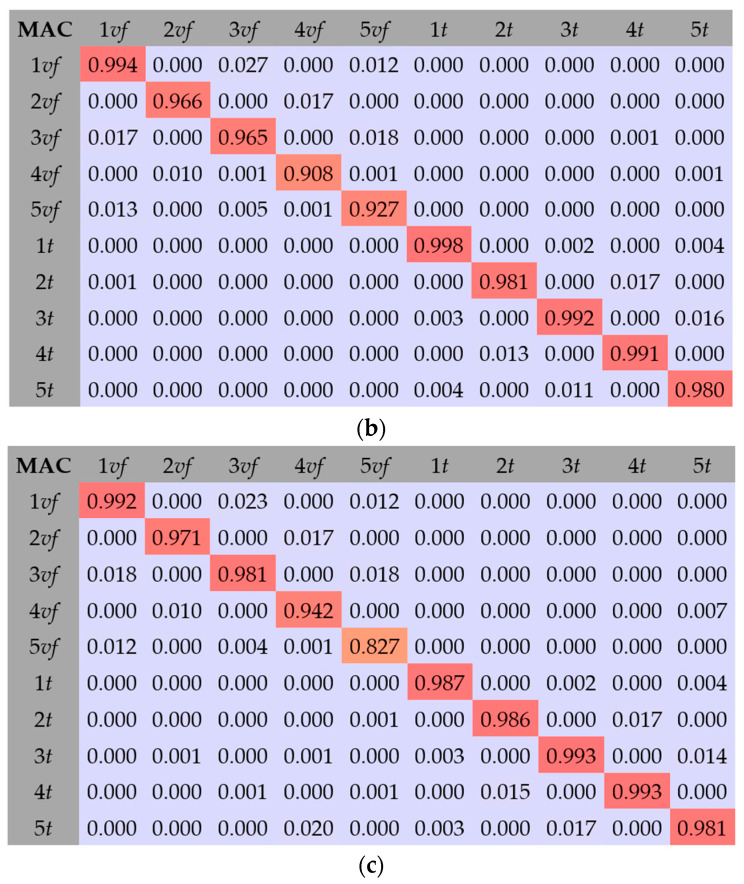
MAC matrix calculations for five flexular and torsional mode shapes for FEM: (**a**) beam C1, (**b**) beam C2, and (**c**) beam C3.

The MAC results for the FEM composite beams C1, C2, and C3 are shown in Figure 12. Figure 13 show MAC matrix calculations for five flexular and torsional mode shapes for the FEM. As can be seen, the results show a high convergence. The MAC coefficient value confirms the correct analyzed mode shapes.

## 4. Discussion

**Figure 14:**
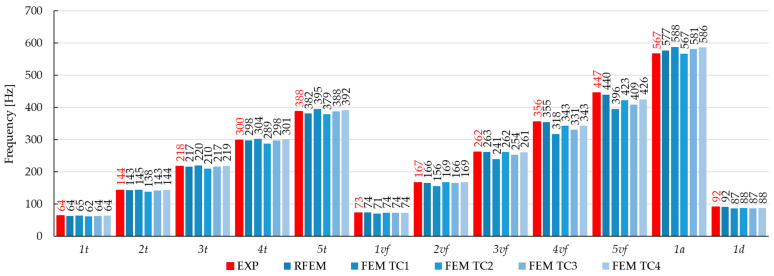
Changes in the natural frequencies for beam C1. The red bars—the experimental results.

**Figure 15:**
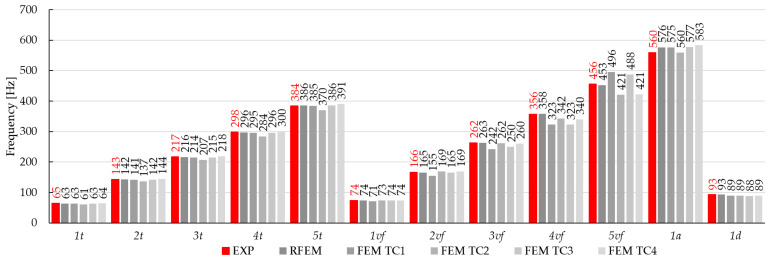
Changes in the natural frequencies for beam C2. The red bars—the experimental results.

**Figure 16:**
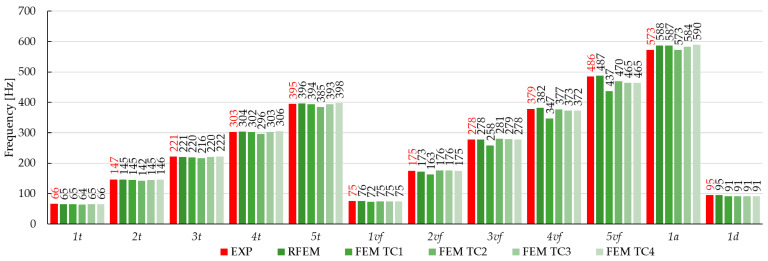
Changes in the natural frequencies for beam C3. The red bars—the experimental results.

**Figure 17:**
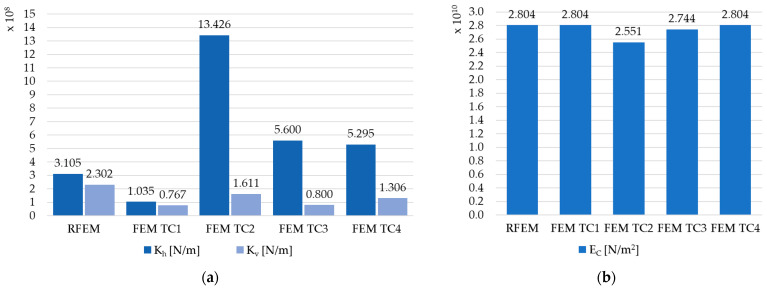
The results in graphic form for beam C1: (**a**) comparison of the equivalent stiffness of the K_h_ and K_v_ connection, and (**b**) comparison of Young’s modulus E_C_.

**Figure 18:**
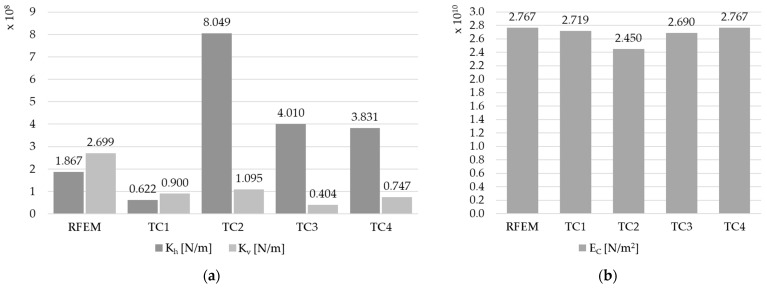
The results in graphic form for beam C2: (**a**) comparison of the equivalent stiffness of the K_h_ and K_v_ connection, and (**b**) comparison of Young’s modulus E_C_.

**Figure 19:**
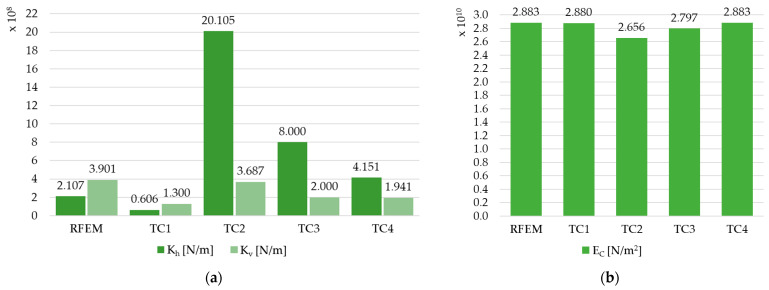
The results in graphic form for beam C3: (**a**) comparison of the equivalent stiffness of the K_h_ and K_v_ connection, and (**b**) comparison of Young’s modulus E_C_.

The main objective of this study was to compare the effectiveness of the finite element method and the rigid finite element method based on the results obtained from the experimental modal analysis carried out on composite beams. This paper focuses on comparing the results from the numerical models and those obtained from experimental tests. The paper presents some of the differences noticed during the analyses, in the general approach to modeling or calculating numerical systems. Summarizing the research presented in this article, the experimental studies show the following:

- The RFEM model provided high agreement with the experimental results. The relative error ranged from −2.5% to 0.5% for the torsional forms of vibration, −1.5% to 1.3% for the flexural, and 1.8% to 2.9% for the axial forms of vibration. The MAC coefficient was in the range of 0.818–0.997. Similar results can be found in the literature [14,17,32,33,34,40];
- The FEM model TC1: Shows the weakest agreement with experimental results, with a relative error ranging from −11.4% to 9.0%. Attempts to linearly recalculate the composite stiffness between the FEM and the RFEM models did not yield satisfactory results;
- The FEM model TC2: Achieved a better fit than TC1, with a relative error of −7.7% to 3.6%. However, the results for the torsional forms were inaccurate, due to underestimation when fitting the axial vibration forms;
- The FEM model TC3: Provided a good fit for the torsional and first two flexural forms (relative error of −0.8% to 0.7%). Weaker results were observed for the next flexural forms, where the relative error ranged from −9.4% to 7.0%;
- The FEM model TC3: Identification with the parameter K_x_ fixed in the first step showed only partial agreement with the experimental results. Small differences in the model results when changing the stiffness parameter of the composite, compared to the expected ones, can also be found in the work [10];
- The FEM model TC4: It had the best agreement with the experimental natural frequencies. The relative error ranged from −1.0% to 1.8% for the torsional forms, −0.1% to 1.6% for the first three flexural forms, and up to 10% for the next flexural forms. Similar relative error values can be found in the works [10,16]. The MAC coefficient was 0.827–0.994, indicating a high convergence with results in the literature [9,10,22,36,41,43];
- Comparing the values of the vibration frequencies between the FEM and the RFEM methods, the range of relative error is −8.66% to 11.40%. For comparison, in the work [14], one can find a greater convergence of results between models, but different engineering systems are modeled. A detailed comparison of vibration frequencies for individual beams is shown Figure 14 (for the beam C1), Figure 15 (for the beam C2), and Figure 16 (for the beam C3), which allows a better assessment of the differences in results. The red bars on the graph represent the results of the experiment.

- The results for the elastic modulus E_C_ in all models are similar, except for the FEM TC2 model, where a significant reduction in E_C_ and a significant overestimation of the composite stiffness K_h_ were noted. The detailed values are shown in Table 10 and in Figure 17 (for the beam C1), Figure 18 (for the beam C2), and Figure 19 (for the beam C3), to analyze the differences. Differences in composite stiffnesses between different methods for modeling the same beam can also be found in the work [19].

Ultimately, the bending and torsional vibration frequencies obtained from both RFEM and FEM calculations were compared in terms of their similarity. Tabular presentation of results are presented in Table A5 (for the beam C1), Table A6 (for the beam C2), Table A7 (for the beam C3).

## 5. Conclusions

The conclusions on the identification of parameters of numerical models are as follows:

- The conducted studies have shown that the FEM and the RFEM methods allow for obtaining numerical models whose results are consistent with the results of experimental studies. The analyzed natural frequencies for the first five bending, the first five torsional, the first distortional, and the first axial form of vibrations of the FEM TC4 and the RFEM models showed a very good match;
- The differences between the numerical model and the experimental results for torsional frequencies ranged from −2.5% to 0.5% (RFEM) and −1.0% to 1.8% (FEM TC4). The differences between the model and the experimental results for flexural frequencies ranged from −1.5% to 1.3% (RFEM) and −0.1% to 1.6% (FEM TC4);
- In both models, high MAC coefficient values were obtained, i.e., 0.818–0.997 (RFEM) and 0.827–0.994 (FEM TC4), which confirms the reliability of the models;
- The linear calculation of the connection parameters obtained during the RFEM calculations turned out to be too much of an oversimplification during the first FEM TC1 calculations. Such a model did not show sufficient convergence with the results from the experimental tests;
- The best results of the numerical FEM model fitting were achieved in the identification process, in which all parameters having a nonlinear effect on the system operation were simultaneously determined. This approach, used in the FEM TC4 model, provided high agreement with the experimental results, which emphasizes the importance of the comprehensive optimization of dynamic parameters in order to increase the accuracy of modeling;
- The research and analysis carried out show that the proposed methodology for modeling the dynamic properties of steel–concrete composite beams makes it possible to obtain calculation results of high reliability, both qualitatively and quantitatively. These methods also work well in the case of more complex structures, allowing an accurate analysis of their dynamic properties, which is important in the design of modern engineering structures.

Conclusions about the differences between the models and their practical consequences are as follows:

- The RFEM is based on rigid finite elements, which allows for simplifying the analysis and reducing the number of degrees of freedom. The FEM, on the other hand, uses a more detailed approach, using deformable elements, which provides greater precision in representing the behavior of the structure, especially in the case of complex geometry and element interactions;
- The finite element division in the FEM model defined 1632 elements on the plate and 544 on the beam, while in the RFEM model, the number of elements depended on the beam: 184 for beam C2 and 264 for beams C1 and C3;
- For the RFEM model, the calculation time turned out to be much shorter—about 20 s, while for the FEM it was about 58 s, for one model recalculation including the time needed to run Abaqus/CAE 2022 (in the background), and for modeling, calculations, and processing the results in Matlab (R2020b);
- The RFEM model showed computation time savings of about 80% compared to the TC4 FEM, due to its simplified structure and lower number of degrees of freedom;
- The identified FEM models allow for more results due to a larger number of finite elements;
- In the RFEM model, the key parameter responsible for the first distortive vibration mode was the K_R,x_ parameter, representing the equivalent stiffness of the connection in the rotational direction, in units of Nm/m. For the FEM model for the modeled Spring elements, the most appropriate was the equivalent stiffness of the joint in the normal direction, denoted by K_x_ (in units of N/m, along the *x*-axis), but this variable was less sensitive to the distortional mode than in the RFEM. A full agreement of this value was not achieved for any FEM model. Finally, for FEM TC4, a relative error for this mode of vibration was obtained in the range of −4.8% to 4.5%, while for RFEM, a perfect fit was obtained;
- In the analyses performed, FEM models were created using a combination of Abaqus/CAE 2022, Python 3.12, and MATLAB (R2020b), which enabled the use of Abaqus/CAE 2022 computational solvers. In contrast, the RFEM method required the development of dedicated software in the MATLAB (R2020b) environment. This difference in approach highlights a greater flexibility, comprehensiveness, and the possibility of using ready-made solutions for FEM, while RFEM requires more work on creating specialized tools adapted to specific analytical needs.

Conclusions about future research and possible applications of the results are as follows:

- The authors want to analyze in more detail the work of FEM numerical models by adding other types of composite definitions, and monitoring the nonlinear dependence of the stiffness of different types of composite in relation to changes in dynamic parameters, in particular, distortional vibration and the bottom flange;
- Analyses are planned to determine the damping parameters in FEM and RFEM models;
- The developed numerical models provide a solid basis for expanding the SHM issue of monitoring the condition of structures and analyzing their dynamic behavior under different loading conditions—which is the basic plan for future research;
- The developed numerical models can form the basis for model expansion and calculations of, for example, bridges, and floors in buildings for residential, public, or industrial use. This will allow the control of the vibrations felt, increasing user comfort;
- The developed models can be used to predict the behavior of structures during earthquakes, especially in the high-frequency range, and various forms of vibrations;
- The identified numerical models provide a very good basis for planning further scientific experiments on the mentioned issues without a lot of work, using the developed numerical algorithms.

## Figures and Tables

**Table 1:** List of geometric and material parameters for beam C1, C2, and C3.

Parameters	Beam C1	Beam C2	Beam C3
Beam weight (kg)	323.75	322.77	326.91
Beam length (m)	3.2	3.2	3.2
Plate width (m)	0.6	0.6	0.6
Poisson’s ratio for concrete slab (-)	0.2	0.2	0.2
Poisson’s ratio for steel (-)	0.3	0.3	0.3
Density of the reinforced concrete slab (kg/m^3^)	2322.1	2309.8	2333.5

**Table 2:** The experimental results for beam C1, C2, and C3.

Mode of Vibration	Beam C1		Beam C2		Beam C3	
	*f_exp_* [Hz]	x [%]	*f_exp_* [Hz]	x [%]	*f_exp_* [Hz]	x [%]
1*t*	64.24	0.26	64.55	0.26	66.35	0.27
2*t*	143.66	0.33	143.06	0.27	146.60	0.26
3*t*	217.68	0.37	216.90	0.35	221.42	0.35
4*t*	299.75	0.36	298.18	0.28	302.83	0.33
5*t*	387.92	0.45	383.97	0.42	394.70	0.46
1*vf*	73.38	0.16	73.59	0.17	75.37	0.14
2*vf*	167.41	0.76	166.03	0.70	174.82	0.48
3*vf*	262.02	0.58	262.24	0.60	277.68	0.49
4*vf*	356.48	0.46	356.25	0.54	378.80	0.46
5*vf*	446.75	0.58	456.09	0.61	485.53	0.47
1*a*	567.30	0.28	559.64	0.46	573.08	0.64
1d	92.04	0.11	92.87	0.06	95.38	0.08

**Table 3:** Number of RFE and SDE obtained for the composite beam C1, C2, and C3.

Beam	Number of RFE			Number of SDE			
	IPE	SLAB	SUM	IPE	SLAB		SUM
				X	X	Y	
C1	33	33 × 7 = 231	264	16	32 × 7 = 224	33 × 6 = 198	438
C2	23	23 × 7 = 161	184	22	22 × 7 = 154	23 × 6 = 138	314
C3	33	33 × 7 = 231	264	32	32 × 7 = 224	33 × 6 = 198	454

**Table 4:** RFEM results.

Mode of Vibration	Beam C1			Beam C2			Beam C3		
	*f_exp_* [Hz]	*f_num_* [Hz]	Δ [%]	*f_exp_* [Hz]	*f_num_* [Hz]	Δ [%]	*f_exp_* [Hz]	*f_num_* [Hz]	Δ [%]
1*t*	64.24	63.75	−0.8	64.55	63.44	−1.7	66.35	64.67	−2.5
2*t*	143.66	143.19	−0.3	143.06	142.47	−0.4	146.60	145.41	−0.8
3*t*	217.68	216.79	−0.4	216.90	215.63	−0.6	221.42	220.58	−0.4
4*t*	299.75	298.27	−0.5	298.18	296.35	−0.6	302.83	303.65	0.3
5*t*	387.92	381.57	−1.6	383.97	385.94	0.5	394.70	395.88	0.3
1*vf*	73.38	74.37	1.3	73.59	73.98	0.5	75.37	75.72	0.5
2*vf*	167.41	165.86	−0.9	166.03	164.85	−0.7	174.82	172.64	−1.2
3*vf*	262.02	262.70	0.3	262.24	262.95	0.3	277.68	278.14	0.2
4*vf*	356.48	355.11	−0.4	356.25	358.02	0.5	378.80	382.43	1.0
5*vf*	446.75	439.87	−1.5	456.09	452.66	−0.7	485.53	487.21	0.3
1*a*	567.30	577.26	1.8	559.64	575.98	2.9	573.08	587.62	2.5
1*d*	92.04	92.04	0.0	92.87	92.87	0.0	95.38	95.38	0.0

**Table 5:** RFEM variable vectors and the index S.

	Beam C1	Beam C2	Beam C3
K_R.X_ [Nm/m]	4.961 × 10^3^	3.717 × 10^3^	2.627 × 10^3^
K_v_ [N/m]	2.302 × 10^8^	2.699 × 10^8^	3.901 × 10^8^
K_h_ [N/m]	3.105 × 10^8^	1.867 × 10^8^	2.107 × 10^8^
E_C_ [N/m^2^]	2.804 × 10^10^	2.767 × 10^10^	2.883 × 10^10^
S [-]	8.89 × 10^−4^	1.32 × 10^3^	1.56 × 10^−3^

**Table 6:** FEM TC1 results with the RFEM vector of decision variables.

Mode of Vibration	Beam C1			Beam C2			Beam C3		
	*f_exp_* [Hz]	*f_num_* [Hz]	Δ [%]	*f_exp_* [Hz]	*f_num_* [Hz]	Δ [%]	*f_exp_* [Hz]	*f_num_* [Hz]	Δ [%]
1*t*	64.24	64.57	0.5	64.55	63.17	−2.1	66.35	64.71	2.5
2*t*	143.66	144.92	0.9	143.06	141.37	−1.2	146.60	144.69	1.3
3*t*	217.68	220.31	1.2	216.90	214.36	−1.2	221.42	219.52	0.9
4*t*	299.75	303.50	1.2	298.18	295.14	−1.0	302.83	302.23	0.2
5*t*	387.92	395.49	2.0	383.97	384.61	0.2	394.70	393.72	0.2
1*vf*	73.38	71.43	−2.7	73.59	70.65	−4.0	75.37	72.47	3.9
2*vf*	167.41	156.33	−6.6	166.03	155.19	−6.5	174.82	163.25	6.6
3*vf*	262.02	240.63	−8.2	262.24	242.16	−7.7	277.68	257.70	7.2
4*vf*	356.48	318.17	−10.7	356.25	323.48	−9.2	378.80	347.33	8.3
5*vf*	446.75	395.64	−11.4	456.09	495.58	8.7	485.53	437.34	9.9
1*a*	567.30	588.21	3.7	559.64	575.38	2.8	573.08	587.27	−2.5
1*d*	92.04	87.04	−5.4	92.87	89.31	−3.8	95.38	90.94	4.7

**Table 7:** FEM TC1 variable vectors and the index S.

	Beam C1	Beam C2	Beam C3
K_x_ [N/m]	1.15 × 10^8^	8.58 × 10^7^	6.06 × 10^7^
K_v_ [N/m]	7.67 × 10^7^	9.00 × 10^7^	1.30 × 10^8^
K_h_ [N/m]	1.04 × 10^8^	6.22 × 10^7^	7.02 × 10^7^
E_C_ [N/m^2^]	2.80 × 10^10^	2.72 × 10^10^	2.88 × 10^10^
S [-]	4.07 × 10^−2^	3.04 × 10^−2^	3.12 × 10^−2^

**Table 8:** FEM TC4 results of the identification process.

Mode of Vibration	Beam C1			Beam C2			Beam C3		
	*f_exp_* [Hz]	*f_num_* [Hz]	Δ [%]	*f_exp_* [Hz]	*f_num_* [Hz]	Δ [%]	*f_exp_* [Hz]	*f_num_* [Hz]	Δ [%]
1*t*	64.24	64.29	0.1	64.55	64.21	−0.5	66.35	65.48	1.3
2*t*	143.66	144.07	0.3	143.06	143.70	0.4	146.60	146.38	0.2
3*t*	217.68	218.87	0.5	216.90	218.10	0.6	221.42	222.18	−0.3
4*t*	299.75	301.35	0.5	298.18	300.16	0.7	302.83	305.73	−1.0
5*t*	387.92	392.47	1.2	383.97	390.78	1.8	394.70	397.95	−0.8
1*vf*	73.38	73.99	0.8	73.59	74.08	0.7	75.37	74.75	0.8
2*vf*	167.41	168.59	0.7	166.03	168.70	1.6	174.82	174.84	0.0
3*vf*	262.02	261.13	−0.3	262.24	259.94	−0.9	277.68	278.05	−0.1
4*vf*	356.48	343.44	−3.7	356.25	340.45	−4.4	378.80	372.28	1.7
5*vf*	446.75	425.54	−4.7	456.09	421.50	−7.6	485.53	464.14	4.4
1*a*	567.30	586.38	3.4	559.64	583.49	4.3	573.08	578.60	−1.0
1*d*	92.04	87.635	−4.8	92.87	89.12	−4.0	95.38	91.11	4.5

**Table 9:** FEM TC4 results of decision variable vectors and the individual identification criteria.

	Beam C1	Beam C2	Beam C3
K_X_ [N/m]	9.10 × 10^8^	8.10 × 10^7^	3.64 × 10^8^
K_v_ [N/m]	1.31 × 10^8^	7.47 × 10^7^	1.71 × 10^8^
K_h_ [N/m]	5.29 × 10^8^	3.83 × 10^8^	6.00 × 10^7^
E_C_ [N/m^2^]	2.80 × 10^10^	2.77 × 10^10^	2.883 × 10^10^
S [-]	7.142 × 10^−3^	1.16 × 10^−2^	4.58 × 10^−3^

**Table 10:** The results of the stiffness of the connection model for RFEM and FEM.

RFEM Parameter	RFEM Model	FEM Parameter	FEM Model			
			TC1	TC2	TC3	TC4
Beam C1						
K_h_ [N/m]	3.11 × 10^8^	K_h_ [N/m]	1.15 × 10^8^	9.06 × 10^8^	5.60 × 10^8^	5.29 × 10^8^
K_v_ [N/m]	2.30 × 10^8^	K_v_ [N/m]	7.67 × 10^7^	1.61 × 10^8^	8.00 × 10^7^	1.31 × 10^8^
K_R.X_ [Nm/m]	4.96 × 10^3^	K_X_ [N/m]	1.04 × 10^8^	1.34 × 10^9^	5.60 × 10^8^	9.10 × 10^8^
E_C_ [N/m^2^]	2.80 × 10^10^	E_C_ [N/m^2^]	2.88 × 10^10^	2.55 × 10^10^	2.74 × 10^10^	2.80 × 10^10^
S [-]	8.90 × 10^−4^	S [-]	4.06 × 10^−2^	7.63 × 10^−3^	1.66 × 10^−2^	7.14 × 10^−3^
Beam C2						
K_h_ [N/m]	1.87 × 10^8^	K_h_ [N/m]	6.22 × 10^7^	8.05 × 10^8^	4.01 × 10^8^	3.83 × 10^8^
K_v_ [N/m]	2.70 × 10^8^	K_v_ [N/m]	9.00 × 10^7^	1.10 × 10^8^	4.04 × 10^7^	7.47 × 10^7^
K_R.X_ [Nm/m]	3.72 × 10^3^	K_X_ [N/m]	8.58 × 10^7^	8.65 × 10^7^	8.65 × 10^7^	8.10 × 10^7^
E_C_ [N/m^2^]	2.77 × 10^10^	E_C_ [N/m^2^]	2.72 × 10^10^	2.45 × 10^10^	2.69 × 10^10^	2.77 × 10^10^
S [-]	1.32 × 10^−3^	S [-]	3.05 × 10^−2^	1.18 × 10^−2^	1.99 × 10^−2^	1.16 × 10^−2^
Beam C3						
K_h_ [N/m]	2.11 × 10^8^	K_h_ [N/m]	6.06 × 10^7^	2.01 × 10^9^	8.00 × 10^8^	4.15 × 10^8^
K_v_ [N/m]	3.90 × 10^8^	K_v_ [N/m]	1.30 × 10^8^	3.69 × 10^8^	2.00 × 10^8^	1.94 × 10^8^
K_R.X_ [Nm/m]	2.63 × 10^3^	K_X_ [N/m]	7.02 × 10^7^	6.00 × 10^7^	6.00 × 10^7^	6.23 × 10^7^
E_C_ [N/m^2^]	2.88 × 10^10^	E_C_ [N/m^2^]	2.88 × 10^10^	2.66 × 10^10^	2.80 × 10^10^	2.88 × 10^10^
S [-]	1.56 × 10^−3^	S [-]	3.12 × 10^−2^	4.57 × 10^−3^	4.92 × 10^−3^	5.21 × 10^−3^

## Data Availability

The original contributions presented in this study are included in the article. Further inquiries can be directed to the corresponding author.

## List of Abbreviations

FEM	finite element method;
RFEM	rigid finite element method;
SHM	Structural Health Monitoring;
MAC	Modal Assurance Criterion;
RFE	rigid finite element;
SDE	spring-damping elements;
TC1, TC2, TC3, and TC2	type of calculation 1,2,3, and 4 for FEM.

## Appendix A

**Table A1:** FEM TC2 results of identification process.

Mode of Vibration	Beam C1			Beam C2			Beam C3		
	*f_exp_* [Hz]	*f_num_* [Hz]	Δ [%]	*f_exp_* [Hz]	*f_num_* [Hz]	Δ [%]	*f_exp_* [Hz]	*f_num_* [Hz]	Δ [%]
1*t*	64.24	61.98	−3.5	64.55	61.28	−5.1	66.35	63.97	3.6
2*t*	143.66	138.49	−3.6	143.06	136.75	−4.4	146.60	142.49	2.8
3*t*	217.68	209.90	−3.6	216.90	206.88	−4.6	221.42	215.83	2.5
4*t*	299.75	288.80	−3.7	298.18	284.41	−4.6	302.83	296.40	2.1
5*t*	387.92	379.07	−2.3	383.97	370.18	−3.6	394.70	385.11	2.4
1*vf*	73.38	73.60	0.3	73.59	73.47	−0.2	75.37	74.50	1.1
2*vf*	167.41	169.01	1.0	166.03	168.98	1.8	174.82	176.00	−0.7
3*vf*	262.02	261.96	0.0	262.24	261.50	−0.3	277.68	280.99	−1.2
4*vf*	356.48	343.35	−3.7	356.25	342.09	−4.0	378.80	377.06	0.5
5*vf*	446.75	423.01	−5.3	456.09	420.94	−7.7	485.53	469.66	3.3
1*a*	567.30	567.30	0.0	559.64	559.64	0.0	573.08	573.08	0.0
1*d*	92.04	87.76	−4.6	92.87	89.46	−3.7	95.38	91.24	4.3

**Table A2:** FEM TC2 results of decision variable vectors and the individual identification criteria.

	Beam C1	Beam C2	Beam C3
K_x_ [N/m]	9.06 × 10^8^	8.65 × 10^7^	6.00 × 10^7^
K_v_ [N/m]	1.611 × 10^8^	1.10 × 10^8^	3.69 × 10^8^
K_h_ [N/m]	1.343 × 10^9^	8.05 × 10^8^	2.01 × 10^9^
E_C_ [N/m^2^]	2.55 × 10^10^	2.45 × 10^10^	2.66 × 10^10^
S [-]	7.67 × 10^−3^	1.18 × 10^−3^	4.58 × 10^−3^

**Table A3:** FEM TC3 results of identification process.

Mode of Vibration	Beam C1			Beam C2			Beam C3		
	*f_exp_* [Hz]	*f_num_* [Hz]	Δ [%]	*f_exp_* [Hz]	*f_num_* [Hz]	Δ [%]	*f_exp_* [Hz]	*f_num_* [Hz]	Δ [%]
1*t*	64.24	63.68	−0.9	64.55	63.38	−1.8	66.35	65.05	2.0
2*t*	143.66	142.62	−0.7	143.06	141.79	−0.9	146.60	145.25	0.9
3*t*	217.68	216.62	−0.5	216.90	215.19	−0.8	221.42	220.38	0.5
4*t*	299.75	298.24	−0.5	298.18	296.13	−0.7	302.83	303.09	−0.1
5*t*	387.92	388.43	0.1	383.97	385.55	0.4	394.70	394.32	0.1
1*vf*	73.38	73.68	0.4	73.59	73.62	0.0	75.37	74.82	0.7
2*vf*	167.41	166.44	−0.6	166.03	165.38	−0.4	174.82	176.18	−0.8
3*vf*	262.02	254.22	−3.0	262.24	249.66	−4.8	277.68	280.88	−1.2
4*vf*	356.48	331.09	−7.1	356.25	322.89	−9.4	378.80	375.81	0.8
5*vf*	446.75	409.43	−8.4	456.09	487.86	7.0	485.53	467.56	3.7
1*a*	567.30	581.45	2.5	559.64	577.00	3.1	573.08	571.58	0.3
1*d*	92.04	87.116	−5.3	92.87	88.16	−5.1	95.38	91.15	4.4

**Table A4:** FEM TC3 results of decision variable vectors and the individual identification criteria.

	Beam C1	Beam C2	Beam C3
K_x_ [N/m]	9.06 × 10^8^	8.65 × 10^7^	6.00 × 10^9^
K_v_ [N/m]	8.00 × 10^7^	4.04 × 10^7^	2.00 × 10^8^
K_h_ [N/m]	5.60 × 10^8^	4.01 × 10^8^	8.00 × 10^8^
E_C_ [N/m^2^]	2.74 × 10^10^	2.69 × 10^10^	2.797 × 10^10^
S [-]	1.65 × 10^−2^	1.98 × 10^−2^	4.03 × 10^−3^

**Table A5:** The comparison of FEM and RFEM results for beam C1.

Beam C1	EXP	RFEM	FEM Model							
			TC1		TC2		TC3		TC4	
Mode of Vibration	*f_exp_*	*f_num_*	*f_num_*	Δ	*f_num_*	Δ	*f_num_*	Δ	*f_num_*	Δ
	[Hz]	[Hz]	[Hz]	[%]	[Hz]	[%]	[Hz]	[%]	[Hz]	[%]
1*t*	64.24	63.27	64.57	−2.02	61.98	2.07	63.68	−0.65	64.29	−1.60
2*t*	143.66	141.61	144.92	−2.28	138.49	2.25	142.62	−0.71	144.07	−1.71
3*t*	217.68	215.00	220.31	−2.41	209.90	2.43	216.62	−0.75	218.87	−1.77
4*t*	299.75	295.95	303.50	−2.49	288.80	2.48	298.24	−0.77	301.35	−1.79
5*t*	387.92	385.40	395.49	−2.55	379.07	1.67	388.43	−0.78	392.47	−1.80
1*vf*	73.38	73.72	71.43	3.20	73.60	0.16	73.68	0.05	73.99	−0.37
2*vf*	167.41	167.01	156.33	6.83	169.01	−1.18	166.44	0.34	168.59	−0.94
3*vf*	262.02	254.94	240.63	5.95	261.96	−2.68	254.22	0.28	261.13	−2.37
4*vf*	356.48	331.45	318.17	4.17	343.35	−3.47	331.09	0.11	343.44	−3.49
5*vf*	446.75	409.20	395.64	3.43	423.01	−3.26	409.43	−0.06	425.53	−3.84
1*a*	567.30	577.82	588.21	−1.77	567.30	1.85	581.45	−0.62	586.38	−1.46
1*d*	92.04	87.12	87.04	0.09	87.76	−0.73	87.12	0.00	87.64	−0.59

**Table A6:** The comparison of FEM and RFEM results for beam C2.

Beam C2	EXP	RFEM	FEM Model							
			TC1		TC2		TC3		TC4	
Mode of Vibration	*f_exp_*	*f_num_*	*f_num_*	Δ	*f_num_*	Δ	*f_num_*	Δ	*f_num_*	Δ
	[Hz]	[Hz]	[Hz]	[%]	[Hz]	[%]	[Hz]	[%]	[Hz]	[%]
1*t*	64.55	63.44	63.17	0.42	61.28	3.52	63.38	0.09	64.21	−1.21
2*t*	143.06	142.47	141.37	0.78	136.75	4.18	141.79	0.48	143.67	−0.83
3*t*	216.90	215.63	214.36	0.59	206.88	4.23	215.19	0.20	218.10	−1.13
4*t*	298.18	296.35	295.14	0.41	284.41	4.20	296.13	0.08	300.15	−1.27
5*t*	383.97	385.94	384.61	0.35	370.18	4.26	385.55	0.10	390.76	−1.23
1*vf*	73.59	73.98	70.65	4.71	73.47	0.69	73.62	0.49	74.08	−0.13
2*vf*	166.03	164.85	155.19	6.23	168.98	−2.44	165.38	−0.32	168.71	−2.29
3*vf*	262.24	262.95	242.16	8.58	261.50	0.55	249.66	5.32	259.94	1.16
4*vf*	356.25	358.02	323.48	10.68	342.09	4.66	322.89	10.88	340.45	5.16
5*vf*	456.09	452.66	495.58	−8.66	420.94	7.54	487.86	−7.21	421.49	7.40
1*a*	559.64	575.98	575.38	0.10	559.64	2.92	577.00	−0.18	583.48	−1.29
1*d*	92.87	92.87	89.31	3.99	89.46	3.81	88.16	5.34	89.12	4.21

**Table A7:** The comparison of FEM and RFEM results for beam C3.

Beam C3	RFEM	FEM Model							
		TC1		TC2		TC3		TC4	
Mode of Vibration	*f_num_*	*f_num_*	Δ	*f_num_*	Δ	*f_num_*	Δ	*f_num_*	Δ
	[Hz]	[Hz]	[%]	[Hz]	[%]	[Hz]	[%]	[Hz]	[%]
1*t*	64.67	64.71	−0.06	63.97	1.10	65.05	−0.58	65.55	−1.35
2*t*	145.41	144.69	0.50	142.49	2.05	145.14	0.19	146.46	−0.72
3*t*	220.58	219.52	0.48	215.83	2.20	220.13	0.21	222.28	−0.76
4*t*	303.65	302.23	0.47	296.40	2.44	302.57	0.36	305.73	−0.68
5*t*	395.88	393.72	0.55	385.11	2.80	393.38	0.64	397.74	−0.47
1*vf*	75.72	72.47	4.49	74.50	1.64	74.77	1.28	74.79	1.25
2*vf*	172.64	163.25	5.75	176.00	−1.91	175.70	−1.74	174.99	−1.34
3*vf*	278.14	257.70	7.93	280.99	−1.02	279.35	−0.43	278.13	0.00
4*vf*	382.43	347.33	10.11	377.06	1.42	373.35	2.43	372.47	2.67
5*vf*	487.21	437.34	11.40	469.66	3.74	464.60	4.87	464.64	4.86
1*a*	587.62	587.27	0.06	573.08	2.54	583.66	0.68	589.95	−0.40
1*d*	95.38	90.94	4.88	91.24	4.54	91.10	4.69	91.10	4.70
